# 

**Published:** 1875-05

**Authors:** 


### Contents of May Number.

### ORIGINAL DEPARTMENT.

ART. I.—The True Uterine Mucous Membrane, its Structure, Functions, and Morbid States. By E. N. Chapman, M. D., Brooklyn, N. Y...... 361

ART. II.—Is Phthisis Hereditary. By Wm A. Bennett, M. D., of Syracuse 381

### MEDICAL NOTES.

ART. I.—Notes on Obstetrics and Gynæcology. By. E. N. Brush, M. D... 389

### EDITORIAL DEPARTMENT.

The American Medical Association and Medical Education,.............. 393
The Buffalo General Hospital,.................................... 396
Erie County Medical Society and Preliminary Education,................. 397
Sugar Coated Pills,............................................ 398
Items,............................................................ 400
Books and Pamphlets Received,.................................... 400

